# Skin‐Interfaced Therapeutic Patches for Wound Fluid Management and Transdermal Drug Delivery

**DOI:** 10.1002/adhm.202504450

**Published:** 2025-11-30

**Authors:** Dongjun Han, Donghyun Kim, Haram Lee, Dong‐Wook Park, Sung Soo Kwak, Joohee Kim

**Affiliations:** ^1^ Bionics Research Center Biomedical Research Division Korea Institute of Science and Technology Seoul Republic of Korea; ^2^ School of Electrical and Computer Engineering University of Seoul Seoul Republic of Korea; ^3^ Center For Semiconductor Research University of Seoul Seoul Republic of Korea; ^4^ Trans Bio Lab Co. Ltd. Seoul Republic of Korea; ^5^ Division of Bio‐Medical Science and Technology, KIST School University of Science and Technology Seoul Republic of Korea

**Keywords:** iontophoresis, therapeutic patch, transdermal drug delivery, wearable, wound fluid

## Abstract

Delayed or non‐healing wounds remain a considerable medical challenge owing to infection‐related complications. Consequently, promoting wound healing through effective dressings and transdermal drug delivery systems is crucial. However, current approaches are hindered by complex drug formulations or dependence on bulky external equipment, limiting their practicability and patient convenience. To overcome these limitations, this study presents a skin‐interfaced therapeutic patch that integrates wound fluid management with a transdermal drug delivery system. The wound fluid management component consists of a microfluidic channel and a chamber combined with a hydrogel film, which collects and preserves wound fluid rich in wound‐healing factors, thereby maintaining optimal hydration and providing a protective barrier. The transdermal drug delivery system utilizes an iontophoresis module incorporating bio‐derived DNA polymers that promote tissue regeneration, enabling the direct and efficient delivery of therapeutic agents through the skin. The multifunctional patch is validated via in vitro and in vivo studies, thereby demonstrating accelerated and robust tissue regeneration.

## Graphical abstract

This study presents an integrated skin‐interfaced device combining microfluidics, hydrogel film technology, flexible electronics, and iontophoresis‐based transdermal delivery of PDRN to enhance wound healing. The device effectively manages wound fluid, maintains optimal moisture, and non‐invasively delivers therapeutic drugs. Both in vitro and in vivo evaluations demonstrate accelerated healing, improved cellular proliferation and migration, and regeneration of healthy skin tissue resembling the original skin.

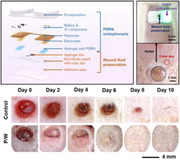

## 1 Introduction

The skin is the largest body organ, providing physical and chemical protection against the external environment [1]. However, it can be damaged by factors such as trauma, burns, and acute or chronic diseases, resulting in wounds [2]. Delayed or non‐healing skin wounds caused by conditions such as diabetes, contamination, or infection may result in severe complications, including necrosis or sepsis [3]. Therefore, protecting wounds from the external environment and enhancing the healing process have become critical medical issues, and numerous studies have been conducted consistently to address these challenges [4, 5, 6, 7].

Conventional wound dressings primarily aim to protect wounds and reduce infection risk. However, traditional dry dressings often cause scab formation and adherence to regenerating tissues. To address these issues, wet dressings, such as hydrogels [8, 9, 10] and hydrocolloids [10, 11, 12], have been widely adopted as alternatives to dry dressings to maintain a moist environment. These dressings preserve wound fluid containing essential healing components [13, 14] and can also deliver therapeutic agents such as antimicrobials, antioxidants, anti‐inflammatory compounds [10, 15, 16] and growth factors [10, 15, 16, 17]. Through these combined functions, they effectively promote wound healing [9, 15, 16, 17, 18]. Nevertheless, drug delivery from hydrogels typically relies on passive diffusion [17, 19, 20, 21, 22], leading to shallow penetration and poor delivery efficiency because of weak driving forces [23, 24, 25, 26].

To overcome the limitations of passive diffusion, several strategies have been proposed. Microneedles enable direct drug transport across the skin barrier [27, 28], while chemical permeation enhancers improve drug permeation rates [29, 30]. However, microneedles are invasive and may cause additional tissue damage, and chemical enhancers require extensive safety validation. Consequently, noninvasive approaches employing external energies, including electricity [18, 31, 32, 33, 34], ultrasound [33, 34, 35], or light [36, 37], have been explored to improve drug delivery efficiency. Among these, ultrasound‐ and light‐based methods require external equipment, making the devices bulky and complex [33, 34, 35, 36, 37]. In contrast, iontophoretic drug delivery employs electrical energy and can be implemented in compact, integrated patches powered by miniaturized electronic circuits [26, 38, 39, 40]. Furthermore, electroosmotic flow enables the transdermal delivery of neutral molecules, broadening the applicability of this approach beyond charged drugs [40, 41].

Despite their advantages, most iontophoretic systems have been restricted to the delivery of small molecules or peptides, as efficient transdermal transport of high‐molecular weight therapeutics remains challenging [42]. In addition, although multifunctional patches integrating drug delivery with wound monitoring have been reported, they have used exudate solely for monitoring rather than exploiting its therapeutic role in wound healing [43, 44]. Moreover, prolonged exposure to exudate can delay chronic wound healing by causing overhydration [45], highlighting the need for drainage once its healing components are depleted.

To address these limitations, we developed a skin‐interfaced therapeutic patch for wound fluid management and transdermal drug delivery. A key feature of this system is the experimentally validated iontophoretic delivery of polydeoxyribonucleotide (PDRN), a high‐molecular weight bio‐derived DNA polymer with well‐documented healing effects [46]. This delivery was enabled by skin appendages such as sweat glands and hair follicles, which provide entry pathways for high‐molecular‐weight compounds across the skin barrier [40, 47]. In addition, the agarose‐glycerol hydrogel preserves wound exudate to maintain healing factors and promote tissue repair [48, 49], while the integrated microfluidic channels drain excess fluid and prevent overhydration. By combining high‐molecular weight drug delivery with therapeutic use of wound exudate, this multifunctional patch demonstrated enhanced healing in both in vitro and in vivo evaluations, highlighting its potential as a cutting‐edge tool for home‐based wound care.

## 2 Result and Discussion

### 2.1 Design of the Integrated Skin‐Interfaced Microfluidic Patch with the Transdermal Drug Delivery System

**FIGURE 1:**
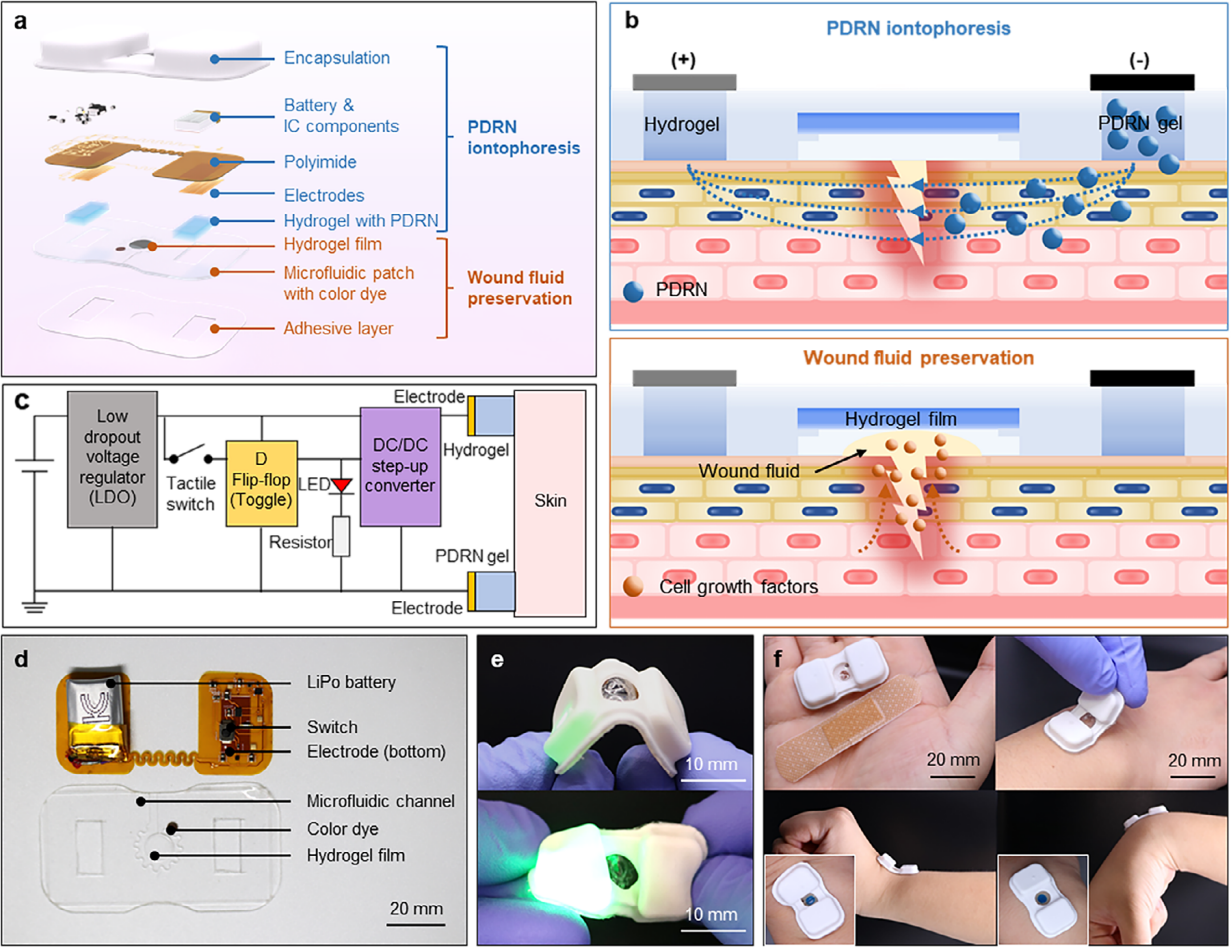
Overview of integrated skin‐interfaced microfluidic patch and transdermal drug delivery system. (a) Illustration of the integrated device. (b) Functions of the integrated device in wound healing: top panel illustrates PDRN iontophoresis and bottom panel demonstrates wound fluid preservation. (c) Block diagram showing the operational scheme of the transdermal drug delivery circuit. (d) Photograph of the microfluidic patch and electrical circuit for the transdermal drug delivery system implemented on a flexible printed circuit board (FPCB). (e) Operational durability and flexibility of the integrated device under physical deformation, including bending with a diameter of 2.5 mm and twisting in various directions. (f) Comparison of a conventional bandage to the integrated device (left), stable adhesion capabilities conforming to curvature (upward: top, downward: bottom), and effective fluid preservation performance (inset images).

As shown in Figure 1a, the integrated system has two primary functions: absorbing and preserving wound fluid, and simultaneously delivering wound‐healing drugs. A microfluidic patch developed using polydimethylsiloxane (PDMS; Sylgard 184, Dow Corning), incorporating a hydrogel film and biocompatible color dye, was designed to collect and preserve the wound fluid while allowing excesses to drain through a microfluidic channel. The transdermal drug delivery system, which performs iontophoresis for wound‐healing drugs, includes an integrated circuit with a battery, electrodes fabricated on a polyimide substrate and a hydrogel pair. In this study, PDRN (Derbiome, SHEBAH BIOTECH Inc.), a bio‐derived DNA polymer that stimulates tissue regeneration and cell growth was used as a wound‐healing drug. The two main components were integrated using an encapsulation layer comprising PDMS doped with 10 wt.% white pigment (Silc Pig, Smooth‐On). A medical‐grade adhesive (1524 skin adhesive, 3 M, Inc.; thickness 60 µm) with openings for inserting a pair of hydrogels (5 × 20 mm) and collecting wound fluid (5 mm in diameter) formed a water‐tight seal and ensured robust adhesion between the device and skin. The cross‐sectional structure of the integrated device is shown in Figure S1.

Figure 1b illustrates the principles of PDRN iontophoresis and wound fluid management for enhanced wound healing. These functions were separated to minimize skin irritation resulting from direct iontophoresis on the wound. PDRN, which promotes cell growth and tissue regeneration, is delivered to the skin via iontophoresis. Applying a voltage generates an electric field between both hydrogels, with one containing PDRN and the other providing ions for pH maintenance and iontophoretic current. Under an applied electric field, the negatively charged PDRN nucleotides migrate toward the positively charged electrode [50, 51], penetrate the epidermis and reach the wound to promote wound healing. The wound fluid containing growth and immune factors was captured using a microfluidic patch and absorbed into the hydrogel film. By preserving this fluid, the wound environment remained moist, allowing the healing components to be retained and delivered back to the wound, thereby promoting wound healing.

The hydrogels in the integrated device were prepared using agarose (Sigma–Aldrich) at different concentrations for specific purposes. As the agarose concentration increased, the pore size within the hydrogel decreased, while hydrogen bonding increased [52]. The increase in the agarose concentration from 1–4% reduced the delivery rate of PDRN from 9.5–3.5 µg/min (Figure S2). Hence, hydrogels for the PDRN delivery were prepared at a relatively low concentration (1 wt.%) to enable the rapid delivery of considerable PDRN. Contrarily, hydrogel moisture retention improved at higher agarose concentrations and after adding glycerol (Figure S3). For example, moisture retention improved by 75% over 12 h at similar agarose and glycerol concentrations. Hence, a hydrogel film for wound fluid preservation was prepared using a 1:1 ratio of water and glycerol with 2% agarose, thereby enhancing moisture retention and physical flexibility.

The iontophoresis circuit for PDRN delivery is configured as shown in Figure 1c. The flexible printed circuit board (FPCB, JLCPCB; thickness: ∼100 µm) is designed in a serpentine shape to enhance mechanical flexibility between the two hydrogel areas and supports surface‐mount electronic components on a gold‐treated surface (∼1 µm), as shown in Figure S4. Herein, a small lithium polymer battery (LiPo battery; 16 × 9 × 4 mm; 3.7 V; 30 mAh) is connected to a low‐dropout voltage regulator (LDO, TPS7A0328PYCHR, Texas Instruments) which stabilizes the output voltage at 2.8 V. This 2.8 V from the LDO is supplied to the input and clock of the D flip‐flop (SN74AUP1G74DQER, Texas Instruments) as well as input of the boost DC/DC converter (TPS61046YFFR, Texas Instruments). When the tactile switch is pressed, the voltage supplied to the clock of the D flip‐flop drops to zero, and rises again when the switch is released. This rising edge toggles the state of the D flip‐flop between Low and High values. This state is then sent to the enable pin of the boost DC/DC converter, thereby outputting a boost voltage of 0. The connected green LED indicates the current operating status based on the D flip‐flop state, as shown in Figure S5. The boost DC/DC converter amplified the voltage to 4–4.2 V, generating an iontophoretic current across the hydrogel pair and skin. With the wet skin resistance at approximately 1 kΩ [53] and hydrogel pair at approximately 15 kΩ, a current of approximately −250 µA was achieved at 4 V (Figure S6). The iontophoretic current is expressed as a negative value based on the cathode‐to‐anode movement of PDRN. This level corresponds with the maximum safe current density (MSCD) for cathodic iontophoresis (−500 µA/cm^2^), considering the hydrogel contact area [38, 40, 54], thereby minimizing the risk of skin irritation. The amplified voltage provides electrical energy for PDRN delivery via a gold‐plated (∼1 µm) electrode on the underside of the FPCB.

As shown in Figure 1d,e, the microfluidic patch was fabricated from stretchable PDMS, while a flexible printed circuit board was fabricated on a flexible polyimide. The device was integrated into a soft silicone‐encapsulating structure which incorporates the components. The integrated device was compact, 40 mm long, 20 mm wide, and 6.5 mm high, and exhibited excellent mechanical properties owing to its flexibility. The integrated device maintained its structural integrity even under bending (radius of curvature: 2.5 mm) and twisting in different directions. The electrical functionality of the iontophoresis circuit remained stable and operational even when bent or twisted, thereby demonstrating its physical durability and operational stability. As shown in Figure 1f, the integrated device was smaller than a standard bandage (72 mm long and 19 mm wide, respectively) and adhered excellently to curved surfaces. It remained securely adhered under bending in different directions, while the fluid preserved in the microfluidic patch was completely sealed, thereby preventing leakage or discharge during free bending.

### 2.2 Wound Fluid Collection and Preservation Characteristics

**FIGURE 2:**
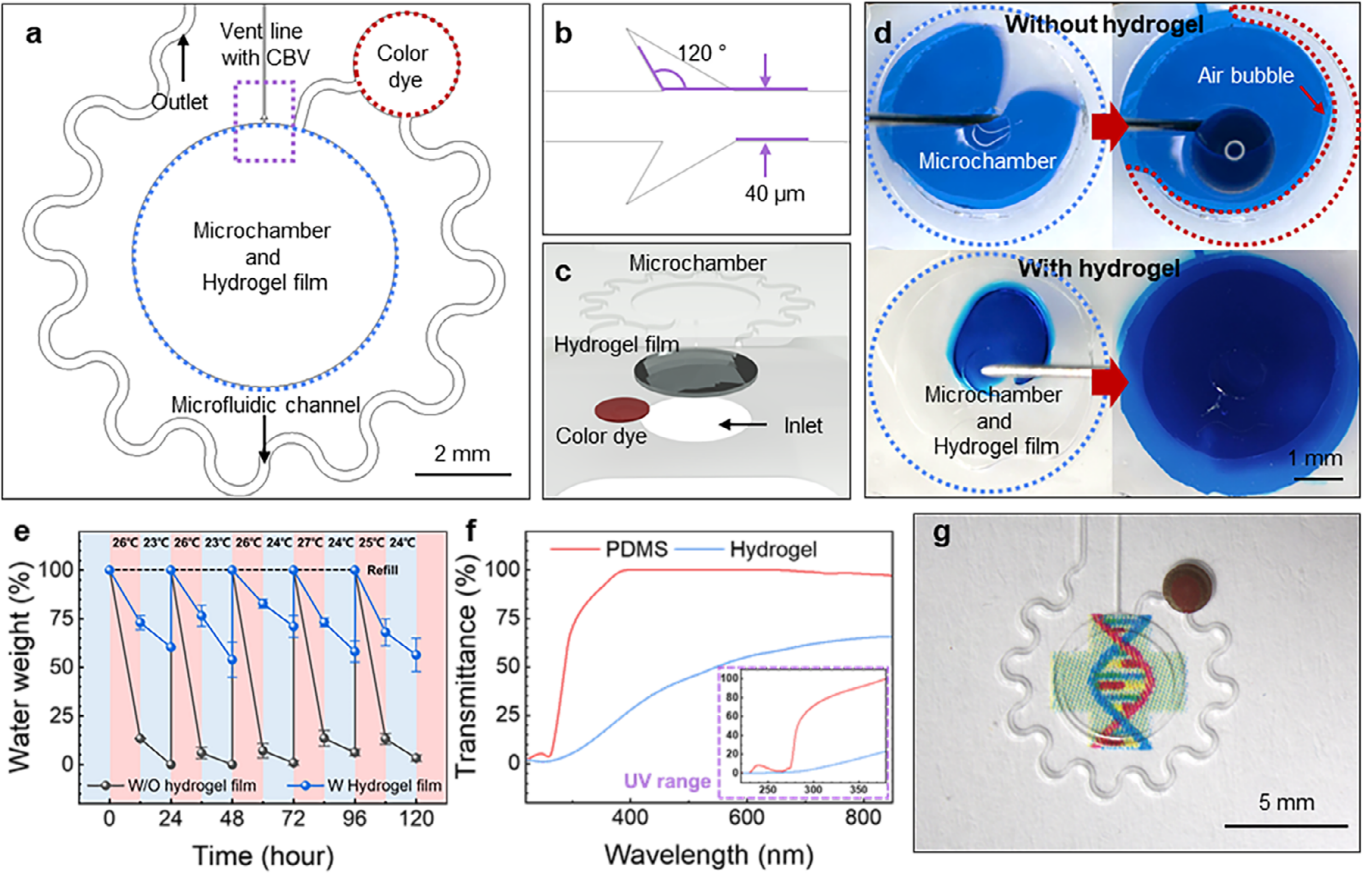
Microfluidic patch for capturing and preserving wound fluid. (a) Schematic illustrating the geometry of the microchamber, hydrogel film, microfluidic channel, color dye, and vent line. (b) Detailed schematic of the vent line featuring a capillary bursting valve. (c) Exploded schematic view depicting the microchamber, hydrogel film, microfluidic channel, and color dye components. (d) Fluid capture comparison within the microchamber, showing results with and without the hydrogel film. (e) Temporal changes in the weight of captured water depending on the presence of the hydrogel film and varying ambient temperature conditions (mean ± SD, n = 5; red represents daytime temperatures above 25°C; blue indicates nighttime temperatures below 25°C). (f) Transmittance characteristics of PDMS and agarose‐glycerol hydrogel measured over a 10 mm optical path (inset displays transmittance in the UV range). (g) Optical transparency demonstration of the assembled microchamber and hydrogel film.

Figure 2 shows the design and functionality of the microfluidic patch developed for wound fluid preservation. As shown in Figure 2a, wound fluid is collected in a microchamber (height: 400 µm; diameter: 5 mm), thereby sealing the wound area securely with an adhesive layer. Inside the microchamber, a protruding support structure (thickness: 100 µm; width: 400 µm; height: 100 µm) provides mechanical stability for the hydrogel film (thickness: 200 µm; diameter: 5 mm). The hydrogel was designed slightly larger than the wound (4 mm) to ensure sufficient exposure of preserved exudate while minimizing the risk of overhydration. The microchamber connects to a vent line (width: 40 µm; height: 200 µm) equipped with a capillary bursting valve with a 120° divergence angle and 40 µm width, as shown in Figure 2b. This capillary burst valve in the vent line has a bursting pressure of approximately 3.7 kPa [55, 56, 57]. According to interstitial fluid dynamics [58], this pressure is sufficient to preserve wound exudate, thereby preventing leakage and allowing the release of internal air. As illustrated in Figure 2c, the wound fluid enters the microchamber via an inlet, wherein it is rapidly absorbed and retained by the hydrogel film. Excess fluid passed through a microfluidic channel coated with a biocompatible colored dye that exited through the outlet. Wound fluid production varies based on the wound type and is in the range of 0.1–1 mL/cm^2^ per day. Given that the internal volume of the microchamber is approximately 7.9 µL (area: 0.25 cm^2^), excess fluid flows out through the microfluidic channel (200 µm width × 200 µm height). As the fluid exits, the colored dye dissolves, thereby indicating fluid flow (Figure S7).

For effective wound healing, consistent and uniform contact must be maintained between the wound surface and captured fluid, which is rich in healing components and free of air bubbles. As shown in Figure 2d, the comparison highlights the difference in the fluid capture with and without the hydrogel film. Without the hydrogel, fluid entering the microchamber at 1 µL/min fills unevenly, thereby trapping air bubbles when the vent line is obstructed. Conversely, the hydrogel film rapidly and uniformly absorbs incoming fluid from the center outwards, thereby effectively releasing air and maintaining a bubble‐free, healing‐rich environment. The dynamic absorption process is further demonstrated in Video S1, which visually illustrates the rapid and uniform uptake of fluid by the hydrogel film. We further evaluated whether the absorbed fluid could be effectively preserved under wet conditions. The patch was immersed in water for 30 min, during which leakage and adhesion stability were assessed. The microfluidic patch remained stable without leakage even under wet conditions (Figure S8).

The hydrogel film considerably improved the moisture retention, particularly after adding glycerol. As shown in Figure 2e, variations in the water weight over time were measured to validate the moisture retention ability [59] including ambient temperature variations owing to daily fluctuations, as the retention period exceeded 24 h. As shown in Figure 2e, microchambers without hydrogel films lose approximately 90% moisture within 12 h above 25°C and dry out completely in the following 12 h below 25°C. Contrarily, chambers with hydrogel films retain an average of 75% moisture after 12 h above 25°C, representing a 375% improvement. The moisture retention remained stable at approximately 60% even after an additional 12 h below 25°C, thereby demonstrating resilience to daily temperature fluctuations. Robust moisture preservation is crucial for ensuring a consistently moist wound environment under varying ambient conditions.

Hydrogel films are advantageous for wound healing owing to their ultraviolet (UV)‐blocking capability. UV exposure during wound healing can result in pigmentation and scar formation [60], thereby making effective UV‐protection crucial. Figure 2f compares the transmittance of PDMS and agarose‐glycerol hydrogel across wavelengths, thereby demonstrating that PDMS transmits more than 40% of the UV radiation above 280 nm, including the harmful UVA and UVB spectra (280–380 nm) [60, 61, 62]. Conversely, the agarose‐glycerol hydrogel exhibited a significantly lower transmittance (less than 20%) across this range. Figure 2g confirms this improved UV‐blocking capability, thereby demonstrating that a microchamber with a hydrogel film provides excellent UV protection than those constructed solely using PDMS.

### 2.3 Safety and Delivery Characteristics of PDRN using Iontophoresis

**FIGURE 3:**
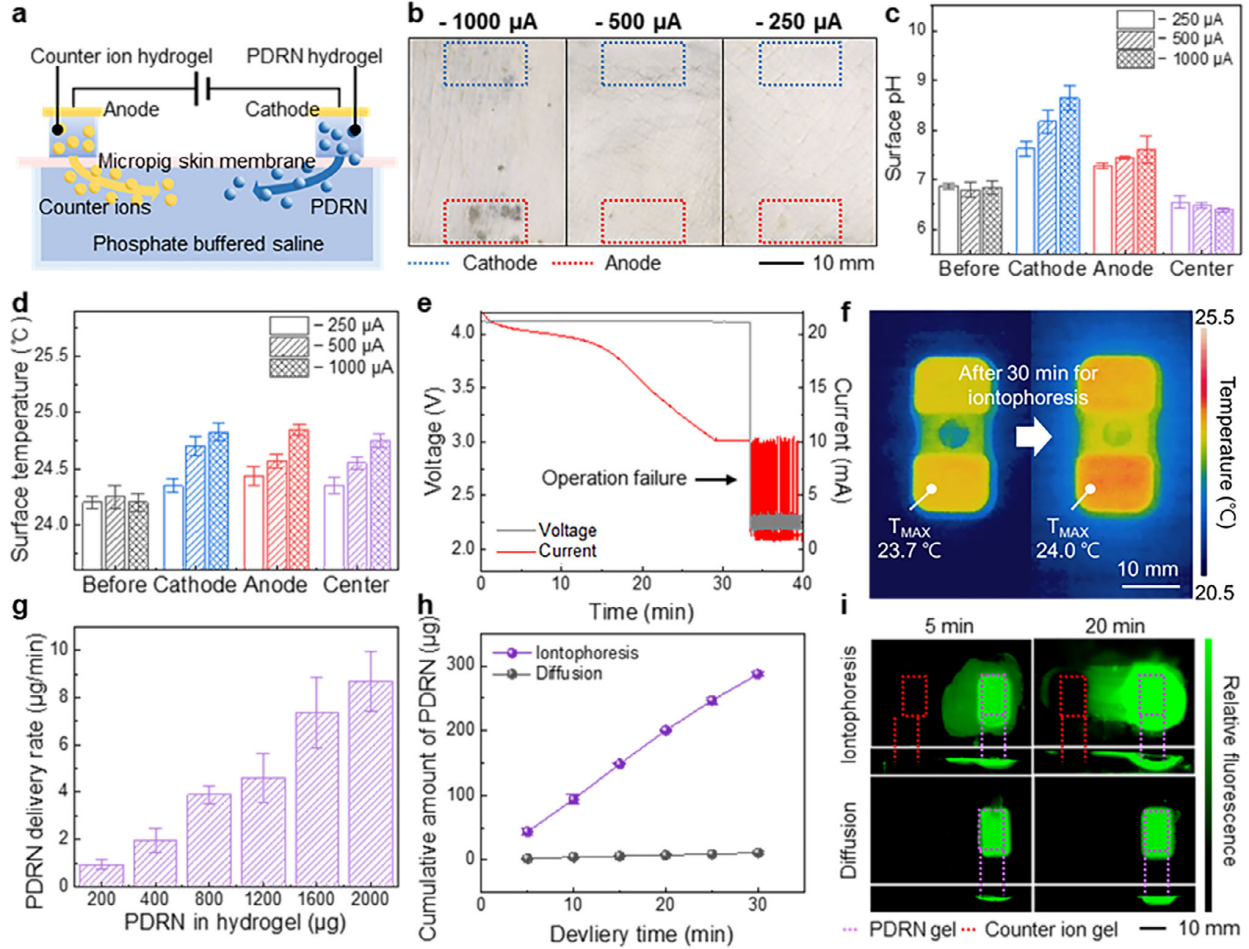
Analysis of Skin irritation, iontophoresis circuit stability, and PDRN delivery. (a) Cross‐sectional view of the iontophoresis experimental model for PDRN delivery. (b) Photographs showing micropig skin membrane changes after 10 min of iontophoresis at various iontophoretic currents. (c) Surface pH variations of the membrane at different locations after 10 min of iontophoresis at varying iontophoretic currents. (d) Surface temperature variations of the membrane at different locations after 10 min of iontophoresis at varying iontophoretic currents. (e) Voltage and current profiles recorded over time during iontophoresis using the LiPo battery‐powered iontophoresis circuit. (f) Infrared thermal images before and after 30 min of PDRN iontophoresis on porcine skin utilizing the integrated device. (g) PDRN delivery rate per minute at an iontophoretic current of −250 µA, based on varying amounts of PDRN loaded into 0.1 mL hydrogel. (h) Cumulative PDRN delivered over time by −250 µA iontophoresis compared with diffusion from hydrogel containing 2000 µg of PDRN in 0.1 mL. (i) Top and cross‐sectional fluorescent images of nucleic acid‐stained agarose gel illustrating PDRN delivery via −250 µA iontophoresis versus diffusion at 5 and 20‐min intervals. Quantitative data in panels c, d, g, and h are presented as mean ± SD (n = 5).

The transdermal delivery of PDRN using the integrated device was evaluated with a simulated transdermal model, as shown in Figure 3a. Counter ion gel was prepared by dissolving a 4 wt.% agarose in a pH 7 buffer, while the PDRN‐loaded hydrogel was prepared by mixing a 2 wt.% agarose solution with a PDRN solution in a 1:1 ratio (final agarose concentration: 1 wt.%). Both hydrogels were formed in PDMS molds of 10 × 5 × 2 mm dimensions. The counterion gel and PDRN hydrogel were connected to the anode and cathode, respectively, and positioned 20 mm apart on a micropig skin membrane (Apures; thickness: 600 µm, dimensions: 30 × 30 mm). The membrane was in direct contact with phosphate‐buffered saline (PBS; Wisd) and maintained at 25°C in a Petri dish to simulate in vivo conditions. Upon voltage application, an iontophoretic current facilitated the transdermal movement of PDRN into the PBS solution. Although increasing the applied voltage enhances ionic mobility and current, excessive current may result in skin irritation, thereby requiring careful optimization.

Figure 3b shows micropig skin membranes images after 10 min of iontophoresis at −1,000, −500, and −250 µA. Severe damage occurred at −1,000 µA owing to voltages exceeding water decomposition thresholds (1.23 V) [63], while no visible damage was observed at −500 and −250 µA. The membrane surface pH and temperature were measured to determine the skin irritation beyond visual assessment (Figure 3c,d). The initial skin pH (∼6.84) increased near the electrode contact points owing to ionic migration and water decomposition, but decreased slightly at the membrane center (Figure S9). At the anode (pH‐buffered hydrogel), pH increments of 0.41, 0.65, and 0.78 were observed at −250, −500, and −1000 µA, respectively. This slight rise, despite water oxidation, can be explained by rapid proton migration and counter‐ion accumulation [64, 65]. The cathode (PDRN hydrogel) exhibited larger pH increases of 0.75, 1.37, and 1.81 due to limited buffer capacity and hydroxide generation. Under similar conditions, the pH of the central membrane decreased by approximately 0.33–0.45, as electro‐osmotic flow drives proton accumulation [66, 67]. These shifts remained considerably below the natural skin pH variability of approximately 1.7 units (from 4.1–5.8) [68, 69], thereby indicating minimal irritation risk. Regarding temperature, the membrane surface was initially 24.2°C and increased only slightly by 0.15, 0.55, and 0.63°C at −250, −500, and −1000 µA, respectively. These slight temperature changes further indicate that −250 µA poses minimal risk of stimulation. Therefore, although higher current density can increase drug delivery (Figure S10) [70], we employed −250 µA, corresponding to the maximum safe current density (MSCD) [38, 40, 54], to minimize skin irritation.

Further safety assessments evaluated the device operation over time, particularly heat generation. Figure 3e shows the voltage and current profiles of the iontophoresis circuit during PDRN delivery, recorded using a source measurement unit (SMU). Although the output voltage remained stable at approximately 4.02 V, the current gradually decreased. After approximately 33 min, the current dropped sharply to approximately 10 mA, followed by fluctuations (1–10 mA) and a voltage drop to approximately 2.2 V. These changes indicate the operational limits of the charge pump‐based boost converter. Consequently, 30 min was determined as the maximum continuous operational period. Infrared thermal imaging (FLIR T530, Teledyne FLIR) as illustrated in Figure 3f shows temperature variations after 30 min of iontophoresis on pig skin maintained under physiological conditions (PBS temperature: 36.5°C). The battery temperature increased slightly from 23.7–24.0°C (+ 0.3°C), while that of the skin increased slightly from 20.5–21.0°C (+0.5°C), thereby indicating a minimal thermal impact.

To quantify transdermal PDRN delivery, its optical absorbance was measured at 260 nm (Figure S11), which corresponds with the PDRN characteristic absorption [19, 71]. Delivery rates were calculated using standard calibration curves (Figure S12). Under different experimental conditions, the integrated device achieved a maximum delivery of 8.68 µg/min during a 10‐min iontophoresis at −250 µA with 2000 µg PDRN loaded in a 0.1 mL hydrogel (Figure 3g). For over 30 min, iontophoresis delivered a total of 287.25 µg PDRN (average rate: 9.58 µg/min), which was superior to diffusion alone, delivering only 11.18 µg (0.37 µg/min)—representing a 2,567% increase (Figure 3h). Although no guideline has defined the maximum recommended local dose of transdermal PDRN in rodents, the delivered amount of 287.25 µg in rats (170–200 g) is far below the systemic dose commonly reported in the literature (8 mg/kg, intraperitoneal) [46]. Considering that transdermal iontophoresis typically reduces systemic effects compared with administration [72, 73, 74], the dose of 8 mg/kg was used only as a conservative safety reference.

Depth penetration analysis demonstrated the advantages of iontophoresis over diffusion. However, direct in vivo visualization of PDRN distribution was hindered by skin autofluorescence and endogenous signal cross‐interference [75, 76, 77, 78, 79]. Therefore, an agarose gel model was employed to indirectly confirm its relative penetration compared with passive diffusion, and fluorescent imaging of the nucleic acid‐stained gel illustrated the distribution and penetration depth (Figure 3i). After 5 and 20 min, iontophoresis at −250 µA achieved penetration depths of approximately 2.9 mm and > 5 mm, respectively, compared to 1.5 mm and 2.8 mm via diffusion—representing approximately 190% enhancement. Contrary to diffusion, whose penetration reduced over time, iontophoresis increased the delivery depth, thereby penetrating a 5 mm‐thick gel within 20 min (Figures S13 and S14). These results confirm the effectiveness of iontophoresis in the delivery rate and penetration depth compared to conventional diffusion methods.

### 2.4 In Vitro Wound Healing Properties

**FIGURE 4:**
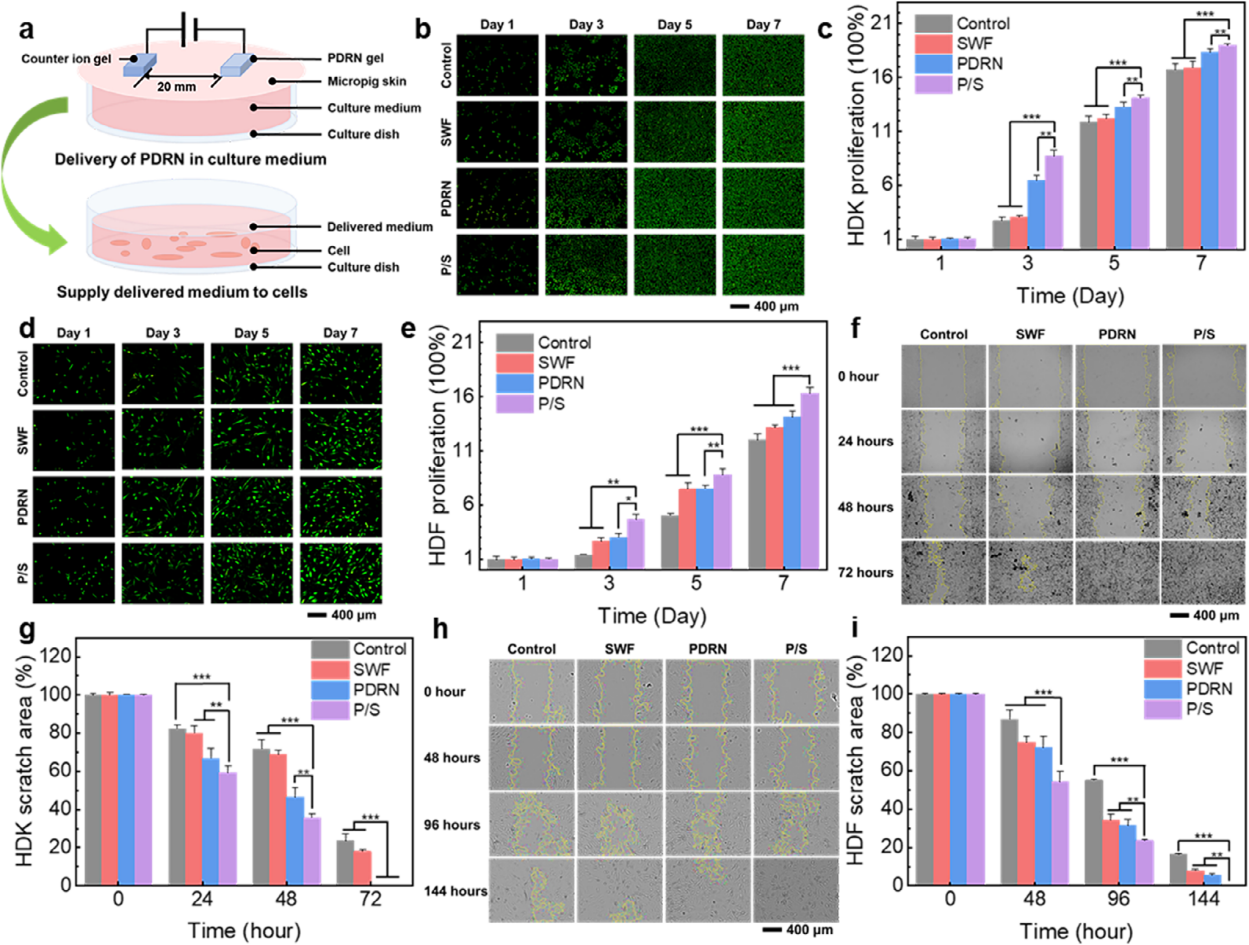
In vitro evaluation of wound healing enhancement effects of the integrated device. (a) Schematic representation of PDRN delivery via iontophoresis into the culture medium. (b) Fluorescence images of HDK (HaCaT) cells from different experimental groups captured at indicated time points. (c) Cell proliferation ratios of HDK cells from each group assessed at indicated time points. (d) Fluorescence images of HDF (CCD‐986Sk) cells and (e) corresponding proliferation ratios from each experimental group recorded at indicated time points. (f) Microscopic images showing scratch assays of HDK cells for each group at specific time intervals. (g) Quantitative analysis of scratch areas in HDK cells from each group across indicated time intervals. (h) Microscopic images of scratch assays performed on HDF cells and i) corresponding quantitative analysis of scratch areas from each group over time. Data represent means ± SD; n = 5. Statistical analysis was performed using one‐way ANOVA with Bonferroni‐corrected post‐hoc *t*‐tests. Statistical significance: ^*^ *p* < 0.05, ^**^ *p* < 0.01, ^***^ *p* < 0.001.

Before validating the in vivo wound healing effectiveness of the integrated device, in vitro studies were conducted to assess the effects of PDRN and simulated wound fluid (SWF) on cell proliferation and migration. Human dermal keratinocytes (HDK, HaCaT) and human dermal fibroblasts (HDF, CCD‐986Sk) were cultured under four conditions: (I) control (standard culture medium), (II) SWF (medium containing 1%), (III) PDRN (delivered via iontophoresis), and (IV) combined treatment (P/S; PDRN and SWF). SWF was prepared by supplementing serum‐free minimum essential medium (MEM) with epidermal growth factor (EGF) and basic fibroblast growth factor (bFGF) at concentrations representative of natural wound fluid [14]. PDRN was delivered using iontophoresis through sterilized micropig skin membranes, thereby providing 50 µg per medium change (Figure 4a).

Cell proliferation was evaluated using live/dead cell staining (calcein‐AM and propidium iodide, MaxView), followed by fluorescence microscopy [36, 80]. Fluorescence imaging of HDK (Figure 4b) exhibited minimal proliferation in the SWF group, which was attributed to the absence of keratinocyte‐specific growth factors, such as the keratinocyte growth factor (KGF). Conversely, the PDRN and P/S groups exhibited enhanced proliferation than the control group. A quantitative analysis (Figure 4c) performed using an image analysis software (ImageJ) revealed that the SWF group exhibited insignificant increases in proliferation (approximately 35%), while the PDRN and P/S groups exhibited considerable increases of 372% and 603%, respectively, than the control group on day 2. Proliferation rates decreased slightly after day 3 owing to limited culture space but remained considerably elevated (140% and 224% on day 5; 160% and 231% on day 7) in the PDRN‐treated groups. Meanwhile, HDF imaging (Figure 4d) demonstrated an increased proliferation across the treated groups compared to the control groups owing to the presence of bFGF in the SWF. The combined P/S treatment resulted in the fastest proliferation, thereby confirming the synergy effects of the PDRN and SWF treatments. The quantitative results (Figure 4e) indicated that proliferation increased in the SWF, PDRN, and P/S groups as follows: day 3 (128%, 163%, and 329%), day 5 (244%, 251%, and 380%), and day 7 (113%, 208%, and 424%). Additionally, fluorescence‐based cell viability assessments confirmed consistent viability across the groups (Figure S15), thereby indicating that neither PDRN nor SWF treatments had adverse effects.

Cell migration was evaluated with scratch assays captured using optical microscopy (Figure 4f). For HDK, enhanced migration in the PDRN and P/S groups facilitated complete scratch closure within 72 h owing to rapid cell doubling [81], while the control and SWF groups retained open scratch areas. The quantitative analysis of the scratch areas (Figure 4g) exhibited minimal improvement (7.5% average) in the SWF group, while the PDRN group exhibited reduced scratch areas—by 19.5% (24 h) and 33.1% (48 h)—compared to control group. The combined P/S group exhibited even greater reductions (27% and 44%, respectively) at the aforementioned intervals. At 72 h, the control group retained 23.6% of the scratch area, while the scratches were completely closed in the PDRN and P/S groups. HDF scratch assays (Figure 4h) demonstrated the effectiveness of the combined treatments despite slower cell doubling [82]. Quantitative analyses (Figure 4i) showed reductions in the scratch areas over each 24‐h interval across the SWF group (5.3–23.8%), PDRN group (6.1–26%) and P/S group (12.1–35.9%) compared to the control group. After 144 h, the control group exhibited an open area of 16.2%, while the P/S group achieved complete closure. The in vitro results confirm the effectiveness of the dual function of the integrated device for wound fluid preservation and PDRN iontophoresis in enhancing cell proliferation and migration. This enhancement promotes accelerated proliferation and epithelialisation, thereby enabling improved wound healing.

### 2.5 In Vivo Wound Healing Properties

**FIGURE 5:**
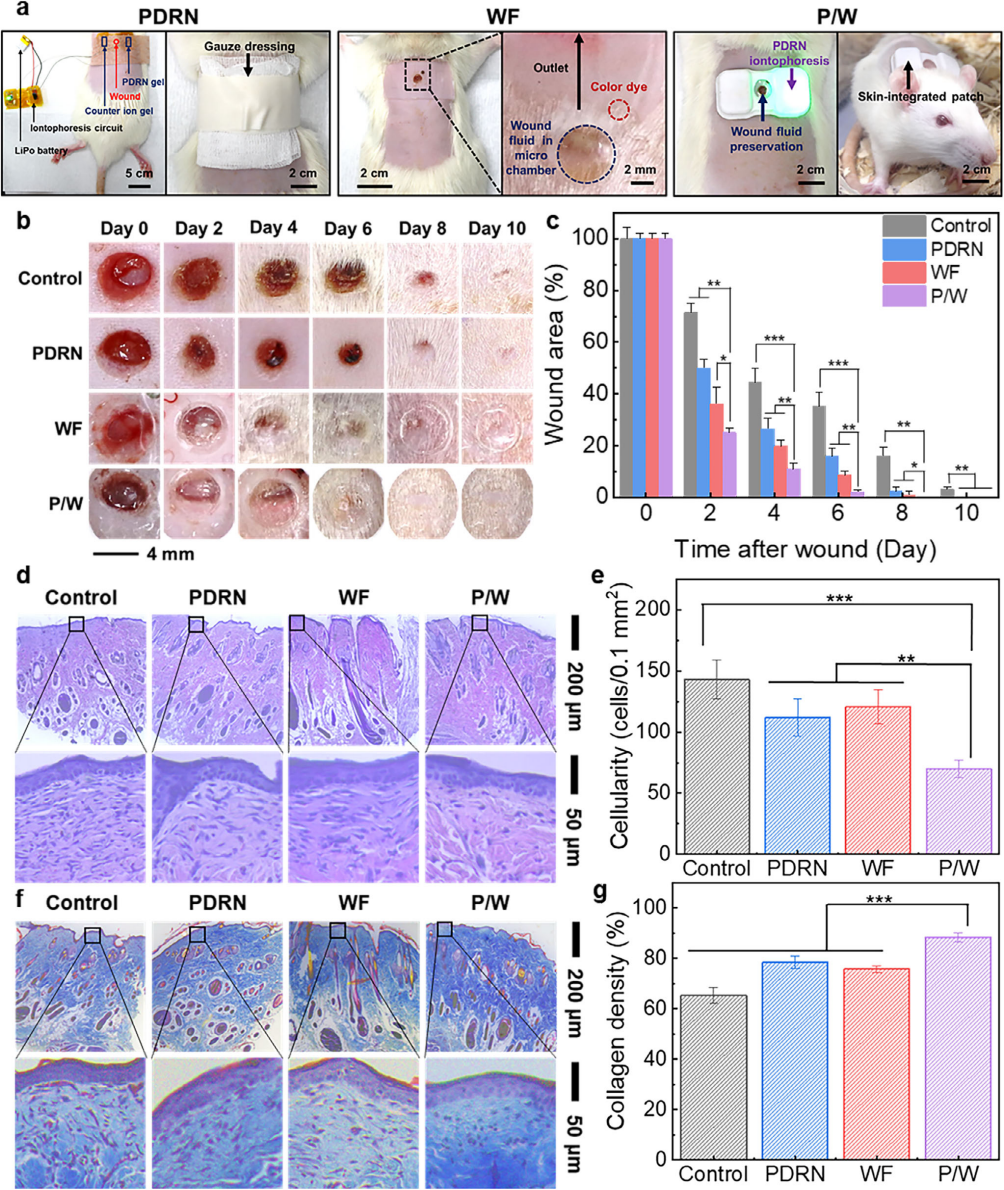
In vivo evaluation of wound healing enhancement effects of the integrated device. (a) Photographs illustrating the treatment procedure and representative post‐treatment images of rats from each experimental group. (b) Representative photographs displaying wound healing progression over time for each treatment group. (c) Quantitative analysis of wound area changes across different treatment groups over time (n = 3 per treatment group). (d) Microscopic images of H&E‐stained skin tissue sections from each treatment group after wound healing, including magnified views. (e) Quantitative analysis of inflammatory cell density in skin tissues post‐wound healing across treatment groups. (f) Microscopic images of MT‐stained skin tissue sections after wound healing for each treatment group, accompanied by magnified images. (g) Quantitative analysis of collagen density in healed skin tissues across treatment groups. Histological analyses were performed with n = 5 per group. Statistical analysis was performed using one‐way ANOVA with Bonferroni‐corrected post‐hoc t‐tests; statistical significance: ^*^ *p* < 0.05, ^**^ *p* < 0.01, ^***^ *p* < 0.0001.

To assess the in vivo wound healing effect of the integrated device, full‐thickness wounds (4 mm diameter) were created on rats (Slc:SD rats, male, 170–200 g, 6–7 weeks, Central Lab. Animal Inc.) using a 4 mm biopsy punch, as previous studies employed wounds of a similar diameter for preclinical wound healing evaluation [83, 84, 85]. The experimental groups were (I) control (gauze dressing only), (II) PDRN (gauze dressing 30 min after PDRN iontophoresis), (III) WF (microfluidic patch for wound fluid preservation) and (IV) P/W (integrated device combining PDRN and WF), as shown in Figure 5a. The therapeutic patches were not replaced during the study unless they were mechanically damaged or detached. They maintained stable adhesion without microchannel occlusion or fouling. No redness, skin irritation, or infection was observed throughout the experimental period (Figure S16). Figure 5b shows wound progression images in each treatment group captured every two days. The PDRN‐treated group demonstrated accelerated wound healing than the control group. However, the dry conditions resulting from the gauze dressing led to scab formation, thereby hampering epithelialization until the scabs detached naturally. Conversely, no scab formation was observed in the WF and P/W groups owing to the moist environment sustained by the microfluidic patch, thereby enabling continuous epithelial regeneration.

Quantitative analyses of the wound area reduction over time (Figure 5c) revealed progressive healing in each group. At two‐day intervals, the control group exhibited wound size reductions of 100%, 71.4%, 44.4%, 35.3%, 16.2%, and 3.2%. The PDRN group demonstrated faster healing, with reductions of 100%, 50.1%, 26.6%, 16.2%, and 2.7%, achieving complete closure by day 8. The WF group demonstrated rapid healing, thereby outperforming the control and PDRN groups (with reductions of 100%, 36.2%, 19.8%, 8.8%, and 1.1%, respectively) and achieving full closure by Day 8. The combined P/W group exhibited the fastest wound healing, with reductions of 100%, 25.1%, 11.0%, and 2.3% achieving complete healing by day 8. These results demonstrate that the two independent modules contributed distinct healing effects and, together, produced a synergistic enhancement of wound healing. In addition, we evaluated the long‐term functional stability of the device using an accelerated aging test (Figure S17). Under eightfold accelerated conditions (60°C, RH ≈ 60%), the iontophoretic voltage remained stable (∼4.11 V), and the current decreased gradually from 21 mA to 16 mA while staying above the operational threshold. The PDRN delivery rate also showed only a modest decline while consistently exceeding passive diffusion. These results indicate that the patch retains effective electrical performance and drug‐delivery capability during prolonged simulated use. Although the present study employed an acute wound model, the demonstrated long‐term device stability, together with previous reports showing that both PDRN and wound exudate are therapeutically effective in chronic wounds [45, 46, 86, 87, 88], suggests that our patch could also be applicable to chronic or delayed‐healing wounds. Moreover, iontophoresis has been reported to remain effective even across larger electrode gaps [89, 90], and the dimensions of the microfluidic chamber can be easily scaled to cover larger wounds. These findings indicate that our therapeutic patch could be effectively applied to larger wounds and also provide healing effects in delayed or non‐healing wounds, highlighting its potential for broader clinical use.

Histological analysis using haematoxylin and eosin (H&E, Abcam) staining provided further insights into the wound healing enhancement effects (Figure 5d). Quantitative analyses of inflammatory cell infiltration per 0.1 mm^2^ (Figure 5e) revealed cell counts of 143, 131, 112, and 70 in the control, WF, PDRN, and P/W groups, respectively. The P/W group exhibited the lowest inflammatory response, with a 49% reduction compared to the control group, indicating an accelerated transition from inflammation to proliferation during healing [91]. Masson's trichrome (MT, Abcam) staining was used to assess collagen deposition and fibrosis after healing (Figure 5f), with the quantified collagen densities shown in Figure 5g. The control, PDRN, WF and P/W groups exhibited collagen densities of 65.2%, 78.3%, 75.7%, and 88.3%, respectively, with the P/W group exhibiting the highest. Collagen deposition is crucial for maintaining skin elasticity and mechanical integrity during tissue remodeling [92]. The superior collagen density (23.1% greater than that of the control group) achieved by the P/W treatment demonstrates the effectiveness of the integrated device in promoting elastic skin tissue regeneration, which resembles the properties of the original, undamaged skin.

## 3 Conclusions

In this study, we developed a skin‐interfaced therapeutic patch that integrates microfluidics, an agarose‐glycerol hydrogel film, iontophoresis, and flexible electronics. The device simultaneously manages wound exudate and enables transdermal delivery of a high‐molecular weight drug (PDRN) to promote effective wound healing. Non‐invasive, low‐irritation iontophoresis achieved more efficient transdermal delivery of PDRN than passive diffusion. The patch incorporates an agarose‐glycerol hydrogel film that preserves wound exudate. The hydrogel keeps the exudate in contact with the wound, supporting natural healing and maintaining a moist, bubble‐free interface. In parallel, the microfluidic channels are designed to drain excess exudate and existing exudate whose healing factors have been depleted, supporting effective wound exudate management.

Across in vitro and in vivo evaluations, our therapeutic patch effectively accelerated wound closure, reduced inflammatory cellularity, and increased collagen density. These results demonstrate that the integration of PDRN iontophoresis and wound exudate management produces synergistic healing effects. Therefore, our therapeutic patch is practically effective for wound care and offers a possible new approach. Moreover, by integrating capabilities such as improved drug permeability, enhanced user convenience, and additional therapeutic modalities (e.g., electrical stimulation, ES), our therapeutic patch could serve as a powerful cutting‐edge tool for home‐based wound care.

## 4 Experimental Section/Methods

### 4.1 Fabrication Process of Integrated Device

The fabrication process of the integrated device was divided into three stages: microfluidic patch fabrication, iontophoresis circuit assembly and device integration. First, a microfluidic patch was fabricated using a silicon wafer mold. A 4‐inch silicon wafer with a thickness of 1 mm was used to form a microfluidic pattern using KMPR 1015 (Kayaku Advanced Materials, Inc.) photoresist deep trench reactive ion etching (DRIE). To enable the PDMS release, the wafer mold was spin‐coated with a thin layer of 950 PMMA A3 (Kayaku Advanced Materials, Inc.) to complete the mold preparation. PDMS, mixed in a 10:1 ratio using an ARE‐310 mixer (THINKY U.S.A., Inc.) is spin‐coated onto the silicon mold, degassed under vacuum and cured at 100°C for 1 h. After curing, the PDMS was removed, coated with a biocompatible red dye and air dried. The non‐patterned PDMS layer was treated using O_2_ plasma and then attached to a 1524 skin adhesive with 5 mm holes. It corresponded with the holes, while 4 mm punch holes were created. This assembly was bonded to the previously fabricated microfluidic PDMS using O_2_ plasma to complete the microfluidic patch fabrication. A 1 µm thick electroless nickel immersion gold (ENIG) treated FPCB (JLCPCB) was applied using Sn42Bi58 low‐temperature solder paste (KEK Original), with a melting point of 138°C. Further, the circuit components were aligned and placed, followed by soldering using a heat gun to form an iontophoresis circuit. The LiPo battery was soldered onto the wires and FPCB using a soldering iron. For the encapsulation layer, Ease Release 200 (Smooth‐On, Inc.) was applied to an aluminum mold fabricated using computerized numerical control (CNC). PDMS containing 1 wt.% white pigment was poured into the mold, degassed under vacuum to remove air bubbles and cured at 150°C for 1 h. After curing, a 9 mm diameter punch hole was created at the center of the encapsulation layer. The iontophoresis circuit was inserted between the fabricated microfluidic patch and encapsulation layer, and they were bonded using O_2_ plasma to integrate the device. The integrated device was completed by laser cutting the outer edges to the desired size.

### 4.2 Characterization of Microfluidic Patch

To prepare the hydrogel film, deionized water and glycerol were mixed in a 1:1 weight ratio, followed by the addition of 2 wt.% agarose. The agarose‐glycerol hydrogel solution was prepared by heating it in a microwave. The prepared solution was poured into a PDMS mold, stored in a sealed container, and refrigerated to form a hydrogel film. The fluid filling characteristics within the microchamber was analyzed by injecting a blue fluid at 10 µL/min using a syringe pump (Fusion200, Chmyx Inc.) while monitoring the process under a low magnification microscope (SZ61, Olympus). The moisture retention with and without the hydrogel film was compared by measuring the weight of the injected water. The weights of the microfluidic patches were measured before and after water injection to calculate the water mass. After sealing the patches—leaving only the outlets and vent lines—their weights were recorded every 12 h. The water was refilled every 24 h, and the process was repeated. Temperature and humidity sensors were placed near the patches to monitor the indoor temperature fluctuations caused by daily temperature variations, with the temperatures periodically recorded.

The PDMS and agarose‐glycerol hydrogel were optically measured using a PMMA cuvette with a light path width and length of 10 × 10 mm (minimum detection wavelength of 220 nm, Brand GMBH). Three milliliters of PDMS and agarose‐glycerol hydrogel, cooled to 50°C, were poured into a cuvette, sealed with a lid and parafilm, and allowed to solidify for 24 h. Before measuring the transmittance of the PDMS and agarose‐glycerol hydrogels, a baseline was established using an empty cuvette. However, transparent images of the microfluidic patch with the hydrogel film inserted were captured using a stereomicroscope (SMZ25, Nikon), while the microfluidic chip was placed on a printed paper featuring the designed image.

### 4.3 Characterization of Iontophoresis using PDRN

PDRN hydrogel was prepared by mixing a 2 wt.% agarose solution (cooled to 70°C) with a PDRN aqueous solution (70°C) in a 1:1 ratio. The counter ion gel was prepared by adding 4 wt.% agarose to pH 7 buffer. PDRN iontophoresis for the skin irritation assessment was conducted using a source measurement unit (SMU, 2460 Keithley, Tektronix). PBS (10 mL) was poured into a Petri dish placed on a hot plate maintained at 25°C, while a membrane sealed waterproof onto the acrylic plate was placed over the Petri dish. Before and after the PDRN delivery, the iontophoretic current was adjusted using the SMU, while the surface pH and temperature at the different membrane locations were measured simultaneously using a skin pH portable meter (HI99181, Hanna Instruments, Inc.). The iontophoretic current powered by the LiPo battery was measured by connecting the FPCB‐assembled circuit to the SMU. The heat generated when operating the integrated device was recorded using an infrared camera (FLIR T530, Teledyne FLIR) while performing iontophoresis on pig skin. Infrared images were captured before and 30 min after iontophoresis, with the images analyzed to determine the changes in the maximum temperature of the integrated device and surrounding pig skin.

The amount of PDRN delivered was calculated by measuring the UV absorbance of the PBS into which PDRN was delivered through the micropig skin membrane. The absorbance of 3 mL PBS containing varying amounts of PDRN was measured in a cuvette. The delivery rate, relative to the PDRN loading, was evaluated after 10 min of iontophoresis. Cumulative delivery was evaluated by measuring the absorbance every 5 min during the delivery process. PBS in contact with the micropig skin membrane was sampled and replaced with fresh PBS. The distribution of PDRN was analyzed using the fluorescent imaging of a 1 wt.% agarose gel containing the nucleic acid stain solution (GreenStar, BIONEER Co.). PDRN was delivered to the stained hydrogel via diffusion and iontophoresis for 5, 10, 15, 20, 25, or 30 min. Fluorescence imaging was performed using a fluorescence imaging system (iBright FL1500, Invitrogen) to capture top‐view and cross‐sectional images. ImageJ was used to measure the height of the fluorescent area to analyze the depth of the PDRN delivery.

### 4.4 Cell Culture using Different Treatment

The HDK cell line HaCaT (RRID: CVCL_0038) was kindly provided by Dr. Youngmee Jung at the Korea Institute of Science and Technology (KIST, Seoul, South Korea) in August 2024 and was confirmed to be free of microbial and mycoplasma contamination. The HDF cell line CCD‐986Sk (RRID: CVCL_4200) was obtained from the Korean Cell Line Bank (KCLB, Seoul, South Korea) on August 2024 and was likewise confirmed to be free of contamination upon arrival. The control group was cultured in the recommended medium for the HaCaT and CCD‐986sk cell lines. HaCaT cells were cultured in DMEM supplemented with 10% FBS and an antimycotic antibiotic (Welgene). CCD‐986Sk cells were cultured in IMDM supplemented with 10% FBS. The SWF group was cultured in 1% SWF and added to the culture medium. SWF were prepared by adding EFG and basic bFGF to MEM at concentrations of 120 and 700 pg/mL, respectively. The PDRN group was cultured in 3 mL of the culture medium containing 50 µg of PDRN delivered via iontophoresis through a micropig skin membrane. The P/S group received SWF and PDRN treatments. All cultures were conducted in an incubator (SANYO Co., Ltd.) at 37°C with 5% CO_2_.

### 4.5 Cell Proliferation Analysis

Cell proliferation rates were analyzed using fluorescence microscopy on days 1, 3, 5, and 7. The cells were transferred to 35 mm diameter culture dishes (SPL Life Sciences) at a density of 3×10^4^ and cultured under control conditions for one day. The culture media were replaced based on the treatment groups, while culturing continued until days 3, 5, and 7. Stained cells were not re‐cultured but maintained separately for each cultivation period, while the culture media of the unstained dishes were replaced on day 4. To prevent serum interference with the staining, the cultured cells were washed thrice using Dulbecco's phosphate‐buffered saline (DPBS; WELGEN Inc.). After mixing DPBS with a serum‐free culture medium, calcein‐AM and propidium iodide were simultaneously added to the cells using MaxView, and the cells were cultured for 20 min. Stained cells were captured using a fluorescence microscope at different focal heights to ensure that all the cells were visible. The captured fluorescent images were analyzed using an ImageJ to determine the area, thereby allowing for the calculation of cell proliferation rates.

### 4.6 Scratch Assay

The scratch assay was conducted by observing cells at 24‐h intervals using an optical microscope. Cells were transferred to a 24‐well plate (SPL Life Sciences) at a density of 3 × 10^4^ and cultured with the recommended culture medium until the area was covered with cells. Once the area was fully covered, a scratch was created at the center using a 200 µL micropipette tip. Cells detached during the scratching process were washed away using DPBS to prevent interference with microscopic observations. Culture media specific to each group were applied, and a similar scratch area was imaged every 24 h using an optical microscope. The culture medium was replaced every 3 d. Images captured using an optical microscope were analysed using ImageJ to calculate the open area, which was expressed as a ratio of the initial area, to determine the contraction rate.

### 4.7 Animal Subjects

All procedures were conducted with the approval of the Institutional Animal Care and Use Committee of the Korea Institute of Science and Technology (KIST‐IACUC‐2024‐056). Slc:SD rats (170–200 g, 6–7 weeks, male) were housed individually to prevent interference with each other's wounds, with ad libitum access to food and water. The rats were maintained on a 12‐h light‐dark cycle (lights off at 8:00 p.m.).

### 4.8 Animal Experiment for In Vivo Wound Healing

Rats were anaesthetized using an inhalation system (Jeungdo Bio & Plant) with oxygen and 3% isoflurane (Hana Pharm). Once anaesthetized, they were placed on a heating pad and 1.5% isoflurane was administered orally to maintain the anaesthesia. The rats were positioned with their bodies fully extended, while their back fur was shaved using a small animal clipper. Hair removal cream was applied for 8 min and later wiped off using a soft wipe soaked in 70% ethanol. To avoid skin irritation, the rats were allowed to recover for one day before wounding. The hair‐removed rats were anaesthetized with isoflurane, placed on a heating pad, and their skin disinfected with 70% ethanol. A full‐thickness wound was created using a biopsy punch, while the blood was removed. To alleviate pain prior to recovery from anaesthesia, the rats were orally administered 1 mL of sterile saline containing acetaminophen (Sigma‐Aldrich) dissolved at a concentration of 15 mg/mL. The control group was dressed with gauze using a medical tape, while the PDRN group underwent 30 min of PDRN iontophoresis and was then dressed with gauze. The WF group applied a sterilized microfluidic patch to the wound site, while the P/W group applied a sterilized integrated device to the wound site and underwent 30 min of transdermal drug delivery of PDRN. All rats were fitted with neck collars to prevent contact with their wound areas and moved to individual cages. Every 24 h after the surgery, the rats were anaesthetized and the wounds photographed under consistent height conditions (∼250 mm above the table) using a camera. The gauze removed from the control and PDRN groups for photography was replaced with a new gauze to prevent infection. After the wound healing process was complete in all groups, skin tissue from a similar location was sampled using a biopsy punch for histological analysis. All the rats were euthanized using a CO_2_ chamber at the end of the wound healing process.

### 4.9 Skin Tissue Histology

Skin tissues collected from rats before and after wound healing were fixed in 4% paraformaldehyde (PFA, GeneAll Biotechnology) diluted in PBS and stored at 2°C. Tissues fixed in 4% PFA for one day were washed for 12 h using tap water, embedded, and processed into paraffin blocks using ethanol, xylene, and paraffin. The prepared paraffin blocks were sectioned into 10 µm slices using a microtome (RM2145, Leica) and transferred onto glass slides using a tissue float bath (Fisher Scientific). The samples were de‐paraffinized and rehydrated using xylene, diluted ethanol, and water, followed by H&E and MT staining. The stained samples were dehydrated, cleaned, and covered with a coverslip containing mounting medium (Mount‐Quick, Hokkaido Sangyo). Images of the prepared samples were captured using an optical microscope and analyzed using image analysis software.

### 4.10 Statistical Analysis

The experimental results are presented as mean ± standard deviation (SD). For comparisons involving more than two groups, one‐way ANOVA followed by Bonferroni‐corrected post‐hoc t‐tests was performed to determine intergroup differences. The analyses were conducted using MS Excel (Microsoft Corporation, Redmond, WA, USA). The sample size (n) for each experiment is indicated in the corresponding figure legend. A p‐value less than 0.05 was considered statistically significant (*p* < 0.05).

## Conflicts of Interest

The authors declare no conflicts of interest.

## Supporting information




**Supporting File 1**: adhm70587‐sup‐0001‐SuppMat.docx.

**Supporting File 2**: adhm70587‐sup‐0002‐VideoS1.mp4.

## Data Availability

The data that support the findings of this study are available from the corresponding author upon reasonable request.
