# Supplementary material for: Skin‐Interfaced Therapeutic Patches for Wound Fluid Management and Transdermal Drug Delivery
Source: Adv Healthc Mater. 2025 Nov 30;15(13):e04450. doi: 10.1002/adhm.202504450 (PMC13058775; doi:10.1002/adhm.202504450)
Supplement: Supplementary file 1 — Supporting File 1: adhm70587‐sup‐0001‐SuppMat.docx. [file ADHM-15-0-s002.docx]

Supporting Information

Skin-interfaced Therapeutic Patches for Wound Fluid Management and Transdermal Drug Delivery

Dongjun Han, Donghyun Kim, Haram Lee, Dong-Wook Park, Sung Soo Kwak*, and Joohee Kim*

**Supporting Display items**


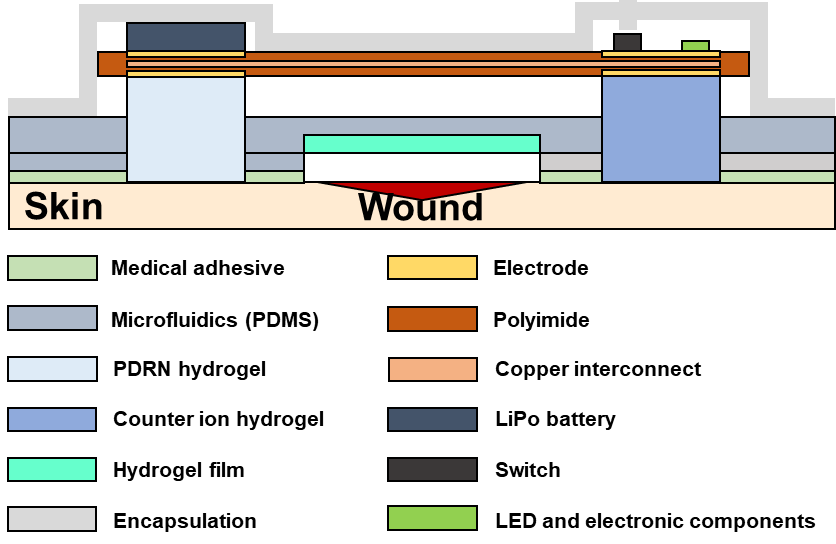


**Figure S1.** Schematic diagram of cross-sectional view of integrated device. Each component is indicated by the color blocks below.

**Figure S2.** Delivery rate of PDRN depending on agarose concentration and glycerol addition (mean ± SD, n = 5).


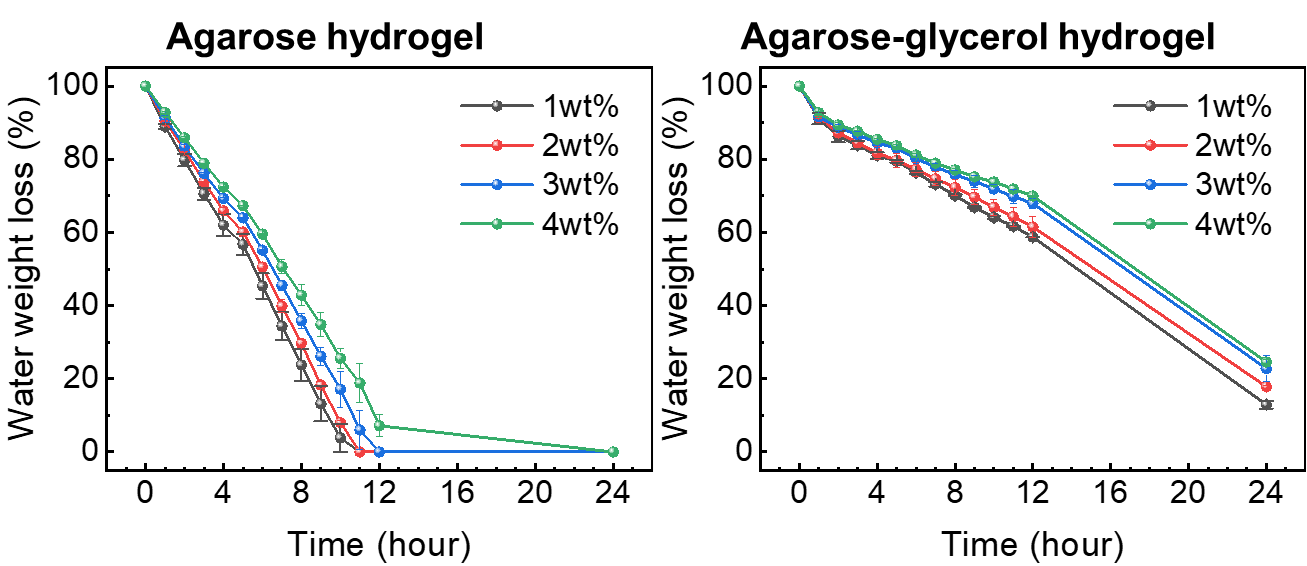


**Figure S3.** The moisture retention rates of agarose hydrogel and agarose-glycerol hydrogel over time (mean ± SD, n = 5).


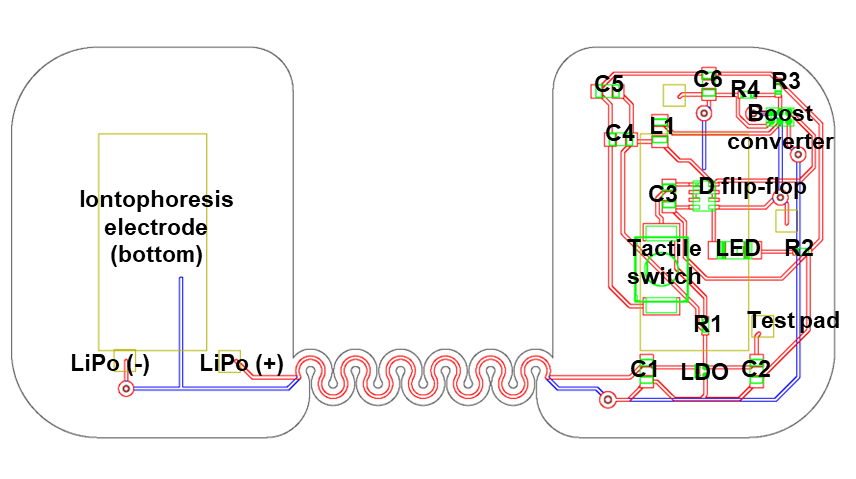


**Figure S4.** Schematic diagram of the iontophoresis circuit. The black lines represent the board edge, the yellow lines indicate the bottom electrode area, the red lines denote connections on the top layer, and the blue lines represent connections on the bottom layer. R stands for resistance, C for capacitance, and L for inductance.


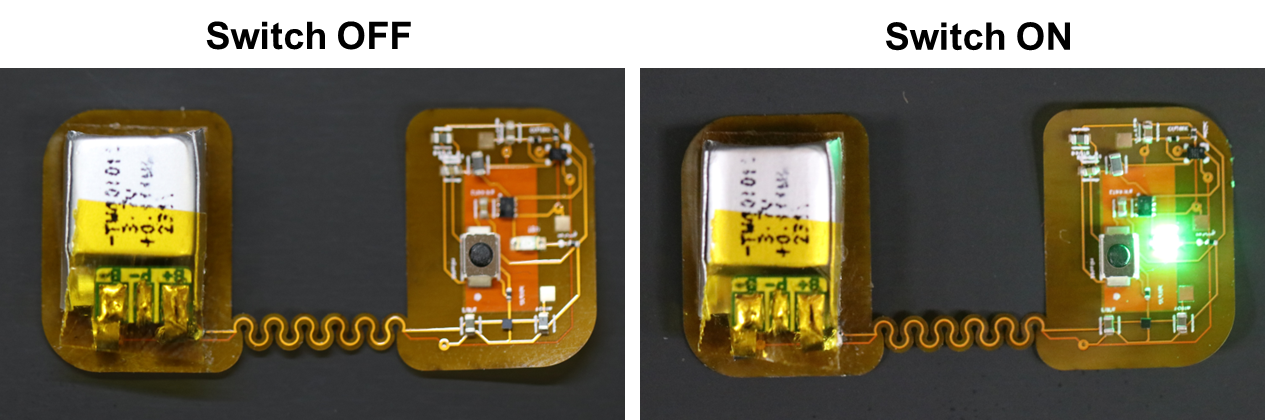


**Figure S5.** Photos of iontophoresis circuit based on output status.

**
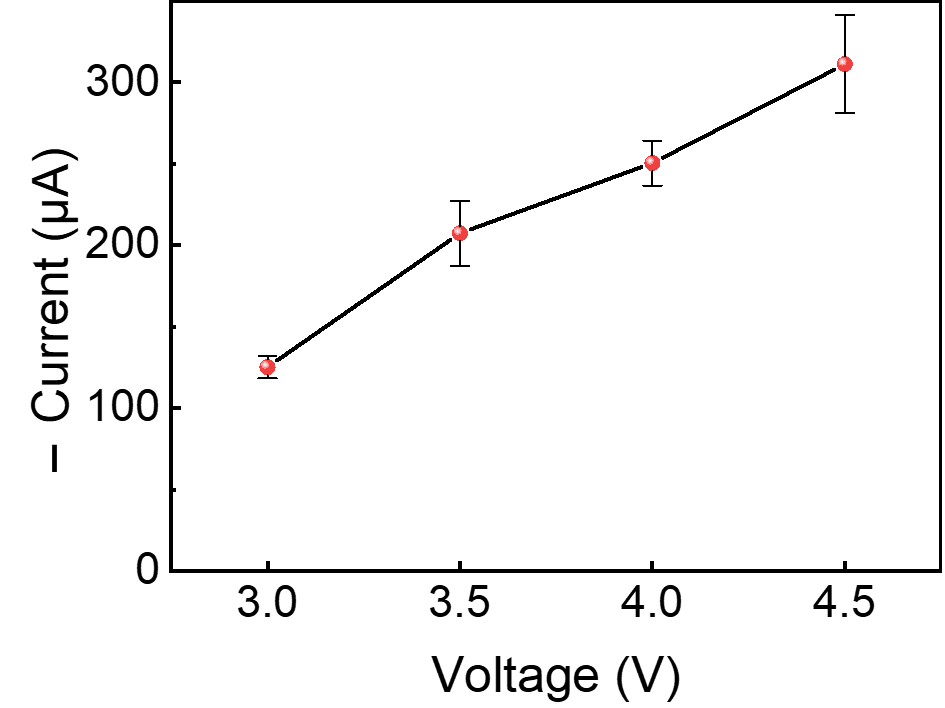
**

**Figure S6.** Iontophoretic current generated by the applied voltage across the hydrogel pair (mean ± SD, n = 5).

**
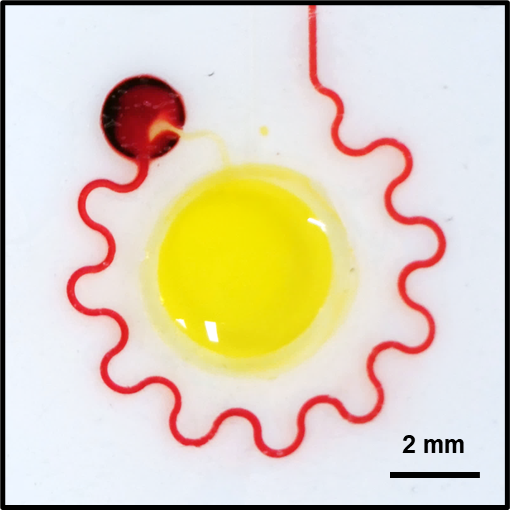
**

**Figure S7.** Microscopy image of fluid flow due to fluid injection into the micro chamber.


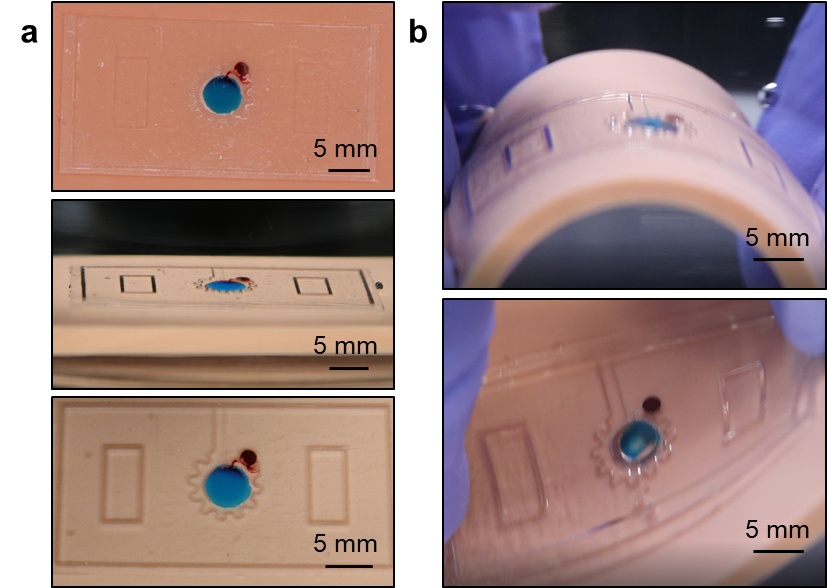


**Figure S8.** Stability of the microfluidic patch in wet conditions. **a** The patch showed no dye leakage after 30 min of water immersion and **b** retained adhesion during mechanical deformation in water.


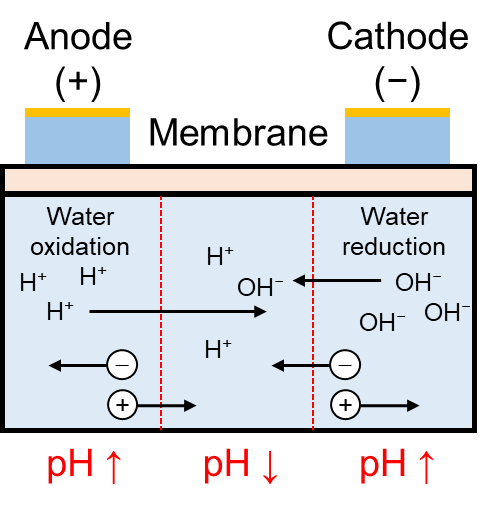


**Figure S9.** Schematic illustration of pH variations across the membrane during iontophoresis.


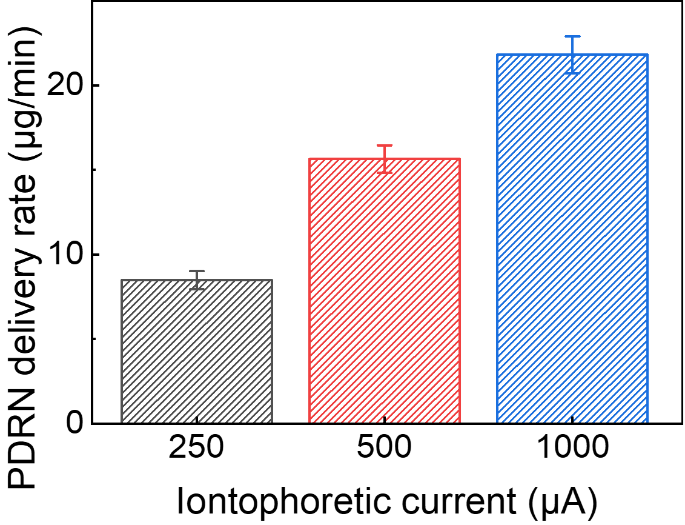


**Figure S10.** PDRN delivery rate according to iontophoretic current (mean ± SD, n = 5).

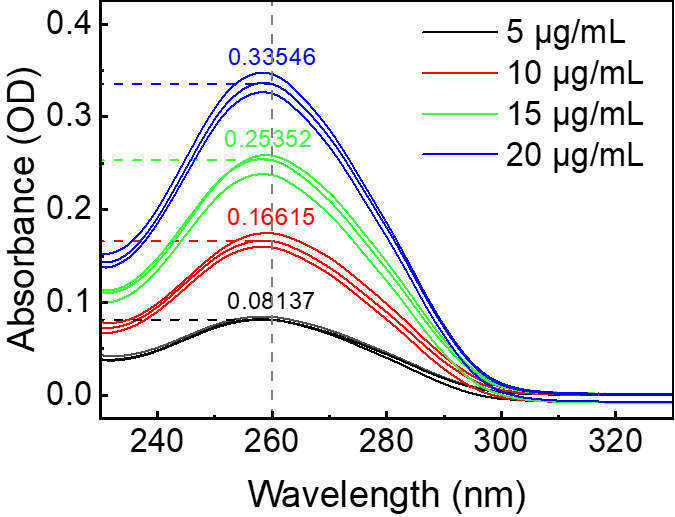


**Figure S11.** UV absorbance according to PDRN concentration in PBS.


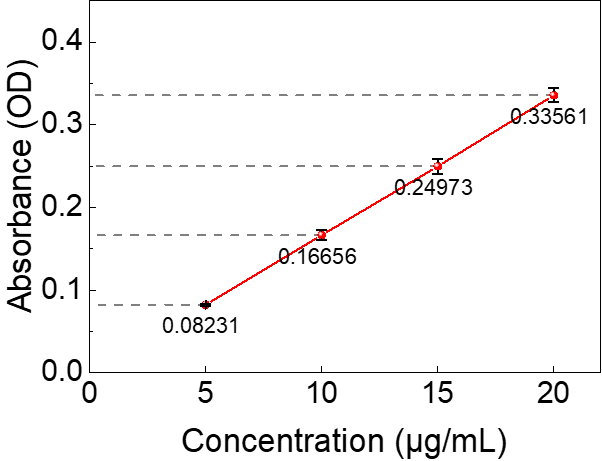


**Figure S12.** Trend of absorbance at 260 nm according to PDRN concentration (mean ± SD, n = 3).

**
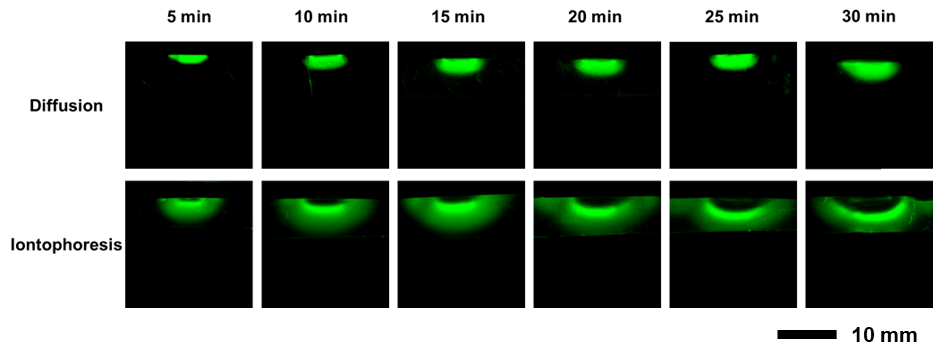
**

**Figure S13.** Cross-sectional fluorescence images of PDRN delivery captured through nucleic acid staining in 1 wt% agarose hydrogel over time.


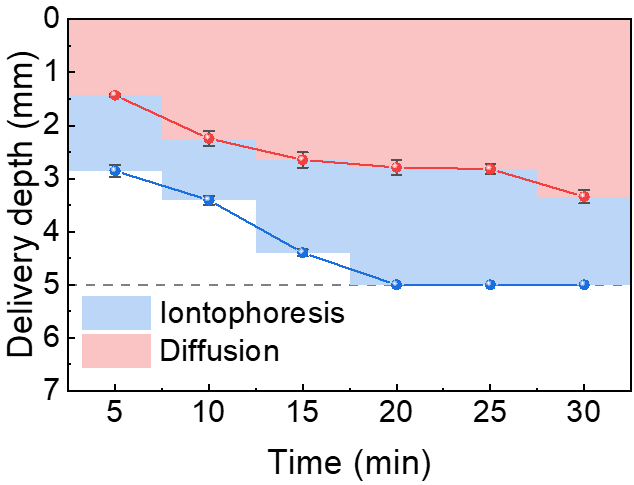


**Figure S14.** Maximum delivery depth of PDRN delivered via diffusion and iontophoresis over time in a 1 wt% agarose gel (mean ± SD, n = 3).


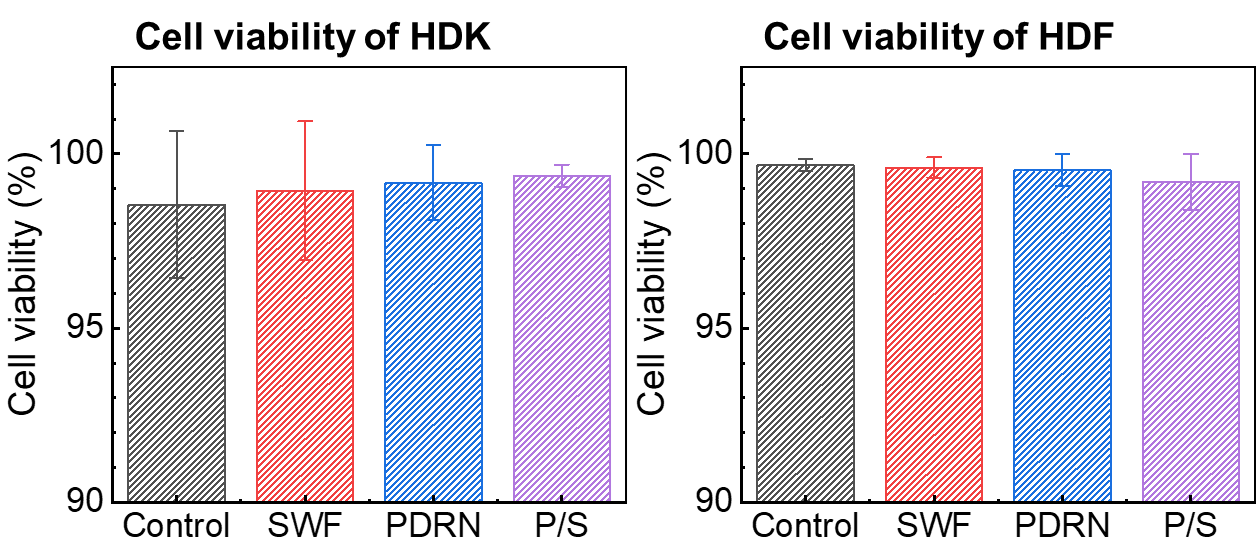


**Figure S15.** Cell viability of human dermal keratinocytes and human dermal fibroblasts for each treatment group assessed by live/dead fluorescent staining (mean ± SD, n = 5).


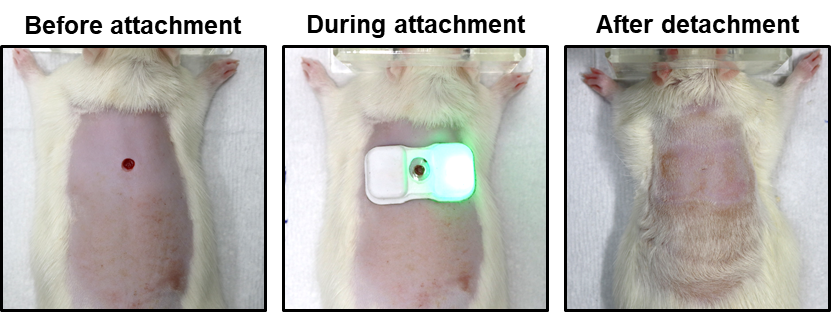


**Figure S16.** Representative photographs of the rat back showing the therapeutic patch before attachment, during attachment, and after detachment.


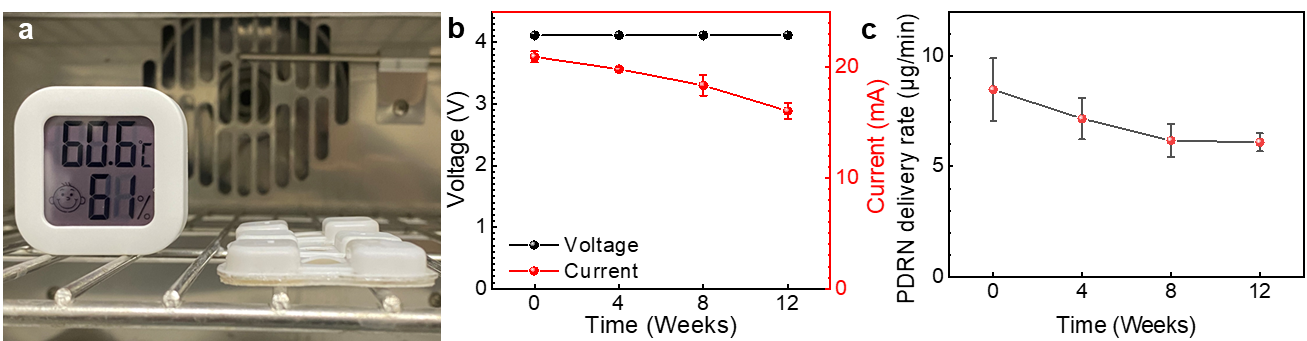


**Figure S17.** **a** Photograph of the accelerated aging test environment. **b** Changes in the voltage and current of the iontophoresis circuit and **c** PDRN delivery rate over time (mean ± SD, n = 3).
